# Use of Electroencephalography for the Study of Gain–Loss Asymmetry in Intertemporal Decision-Making

**DOI:** 10.3389/fnins.2018.00984

**Published:** 2018-12-21

**Authors:** Lei Zhao, Zuoli Shi, Qian Zheng, Huadong Chu, Lin Xu, Fengpei Hu

**Affiliations:** ^1^College of Economics and Management, Zhejiang University of Technology, Hangzhou, China; ^2^Zhijiang College of Zhejiang University of Technology, Shaoxin, China; ^3^School of Humanities and Law, Hangzhou Dianzi University, Hangzhou, China

**Keywords:** intertemporal decision-making, intertemporal choice, event-related potential, graph theory, brain networks, small-worldness index

## Abstract

Intertemporal decision-making refers to the process whereby an individual evaluates and selects among competing alternatives based on the cost and benefit over time. While most previous studies on temporal discounting focused their attention on the gain context, only a few explored the loss context. In the present study, both the event-related potentials (ERPs) and the graph theory analysis were employed to investigate the differences in intertemporal decision-making between the gain and loss frameworks. Our results suggested that participants preferred the short latency/small amount (SS) alternatives and exhibited a smaller discount rate in a loss context compared to a gain framework. Furthermore, our ERP data indicated that the P200 component could constitute a preliminary assessment of the decision-making, related to gain and loss. In contrast, the N2 component was associated with negative emotions and showed significantly bigger amplitudes in the loss context, when compared to the gain framework. Further analyses of brain networks suggested the loss decision-making brain network to have a larger small-worldness index given individuals' loss aversion. Taken together, intertemploral decision-making in a loss context was accompanied by a greater brain response due to the negative emotions linked to loss aversion.

## Introduction

In everyday life, people make trade-off decisions according to varying time constraints and circumstances. For example, an individual needs to select one of two following options when claiming his lottery prize in lottery stores: (1) to receive 90 Chinese Yuan (CNY) immediately or (2) to collect CNY 100 in a month. A large proportion of the people faced with this decision prefers the former option, revealing an unwillingness to wait for an additional month. Specifically, they perceive the immediate value of CNY 90 as larger than that of CNY 100 if received in a month. Successively, the individual will encounter a similar situation when paying his credit card penalties, considering that credit card companies will also offer the option of either paying CNY 90 immediately or CNY 100 in a month. Most of the people will still choose to immediately pay CNY 90, due to their considered higher worth than CNY 100 if collected in a month. However, the following question arises: why would someone show different valuation patterns based on different contexts?

The evaluation and decision-making process that people use to weigh their options according to different time points and events is called intertemporal decision-making (Prelec and Loewenstein, 1991; Frederick et al., 2002), which requires people to choose between short latency/small amount (SS) and long latency/large amount (LL) alternatives. Previous studies found that, compared to SS options, the subjective value of LL alternatives decreases with time, a phenomenon which is referred to as delay discounting (Critchfield and Kollins, 2001; Du et al., 2002; Green and Myerson, 2004; Berns et al., 2007; Scheres et al., 2013). Furthermore, Thaler and Shefrin (1981) described an asymmetry in gain and loss discounting, i.e., subjects discount delayed losses less steeply than gains, also known as the “sign effect.” (Loewenstein, 1988) Therefore, we expect participants to make more choices in the SS range in the loss context, indicating a preference for a more immediate loss rather than a delayed one.

The advancement in brain imaging provided novel statistical approaches to explore the brain mechanism underlying intertemporal decision-making (Green and Myerson, 2004; McClure et al., 2004a,b; Kable and Glimcher, 2007, 2010; e.g., Green and Myerson, 2004; Bickel et al., 2009; Green et al., 2010). For instance, while investigating the brain mechanisms behind the temporal decision of money, juice, and drinking water, McClure et al. (2004a, 2007) reported the asymmetry in the subject's preference for SS and LL to be affected by two distinctive factors, namely valuation and choice (Kable and Glimcher, 2007, 2010; Peters and Büchel, 2011). In addition, Xu et al. (2009) adopted the fMRI technique to compare the cerebral mechanisms underlying this form of decision-making and identified both the lateral prefrontal cortex (LPFC) and the posterior parietal cortex (PPC) to be activated during gain and loss contexts. However, significant differences in their level of activation exist, as higher activation is observed in the loss context compared to gain framework. Moreover, brain regions related to negative emotions (e.g., insula and thalamus) exhibited a greater activation in loss contexts. The authors thus concluded that this suggested the enhanced sensitivity to losses may be driven by negative emotions.

However, only a few studies explored the time course of the brain activity underlying intertemporal decision-making. For example, although Gui et al. (2016) studied the effect of both the reward magnitude and the time delay on intertemporal choices using the ERPs, they only focused on the gain context. Their results indicated that the P200 component may constitute a preliminary assessment of both the reward and time delay, whereas the N2 component is mainly important for the interaction between their valuation (Gui et al., 2016). In the current study, we aimed at comparing the changes in electroencephalography (EEG) under both the gain and loss contexts, specifically through the analysis of ERPs to make a thorough inquiry into the discrepancy between different contexts in intertemporal decision-making. During information processing, the P200 was considered as a quick response and evaluation to stimulus, giving that negative stimuli elicit a higher amplitude than either positive or neutral stimuli (Carretié et al., 2001; Huang and Luo, 2006; Potts et al., 2006; Boudreau et al., 2009; Wang et al., 2012). Moreover, previous studies described that loss aversion occurs in loss decisions and shows greater activation in emotion-related brain regions (Xu et al., 2009). Therefore, we hypothesize that the P200 component will demonstrate different characteristics under the gain and loss conditions. Similarly, the N2 is also a frequently investigated component that was claimed to be reflecting conflict during decision-making, showing higher amplitudes under negative conditions (Nieuwenhuis et al., 2003; Folstein and Van Petten, 2008; Ma et al., 2015). For instance, Gehring and Willoughby (2002) found that the prefrontal lobe would generate a larger N2 when choosing the loss option, as opposed to the gain alternative, in a risky gambling situation. In concordance, other researchers found a valence effect of emotion on the N2 component, and consider it as more attentional resource allocation to negative stimuli in the early stage (e.g., Yuan et al., 2007; Ma et al., 2015). Therefore, we hypothesize that the frontal N2 component will show a greater amplitude in a loss context. Additionally, another important component is the P300, which is widely discussed in the decision-making field and was considered to be associated with attention, evaluation, memory processes, processing capacity and mental workload (e.g., Ford et al., 1982; Polich, 2007). Specifically, given that previous research indicated that decisions about delayed rewards involve more controlled cognitive processes and future-minded memory thinking (McClure et al., 2004a; Peters and Büchel, 2010), we may expect larger P300 amplitudes for SS choices compared to the LL ones.

Recently, graph theory has been widely used to investigate the brain structural and functional connectivity from fMRI, EEG, and MEG data, providing increased information about human cognition compared to simpler univariate approaches which treat each brain region in isolation. Therefore, we also used graph theory in the current study to analyze the brain topological network structure behind SS and LL selection in gain and loss contexts. Graphs are abstract representations of networks and contain sets of vertices (nodes) linked by edges (connections), which can be characterized by both a clustering coefficient C (a measure of the local interconnectedness) and a characteristic path length L (an indicator of the overall integration, see Graph Theoretical Analysis below for details). In fact, it was previously shown that the human brain network can be viewed as a small-world network, in which a node is a brain region and an edge represents a functional correlation between two nodes (e.g., Bassett and Bullmore, 2006). A small-worldness network is a type of graph that combines a strong local clustering and a short characteristic path length (a high C and a short L), which reflects the brain's ability to efficiently integrate information and selection when confronted with complex situations. For instance, a loss of small-worldness could be associated with conditions of reduced consciousness (Uehara et al., 2014). In addition, neuropathology studies reported that patients (e.g., schizophrenia and Alzheimer disease) showed lower small-worldness than healthy people (Stam et al., 2007; Pachou et al., 2008). In contrast, higher small-worldness could reflect the activity coordination between different neural assemblies to accomplish a complex cognitive task. For instance, Micheloyannis et al. (2006) indicated that less educated subjects showed stronger small-worldness than those with a higher education, given the same behavioral performance. Considering that less educated subjects were often characterized by lower cognitive abilities, the higher small-worldness observed may suggest a need for the optimization of their neuronal organization to perform well in demanding cognitive tasks. This compensatory mechanism was also supported by the increased small-worldness found in sleep-deprived subjects (Liu et al., 2015). Further studies also revealed a correlation between emotions and small-worldness properties, i.e., increased small-worldness in more emotion processing situations (e.g., Li et al., 2017). Compared to gain decisions, people require more resources and a more complex information processing to make loss decisions, and cause more negative emotions. Therefore, we hypothesize that, compared to a gain framework, the brain network will show a stronger small-worldness index in a loss context. Furthermore, cortical connections will appear in different brain areas and show different intensity.

## Experimental Procedures

### Participants

Twenty-eight healthy participants who were students at the Zhejiang University of Technology with no separate source of income were recruited in this study. Three of them were excluded as they chose the same option in a gain or loss context, whereas two additional participants were rejected given the high number of EEG artifacts. A total of 23 participants (14 women) aged 18–26 years (M = 21.32, SD = 2.25) were analyzed. All participants with normal or correct-to-normal vision and had no history of neurological or psychiatric disease or brain damage. They were all right-handed and could skillfully operate computers.

### Ethics Statement

All procedures were carried out with the adequate understanding and written consent of the participants. The participants were reminded of their right to discontinue participation at any time. The Research Ethics Board of Zhejiang University of Technology approved all procedures.

### Procedures

**Figure 1:**
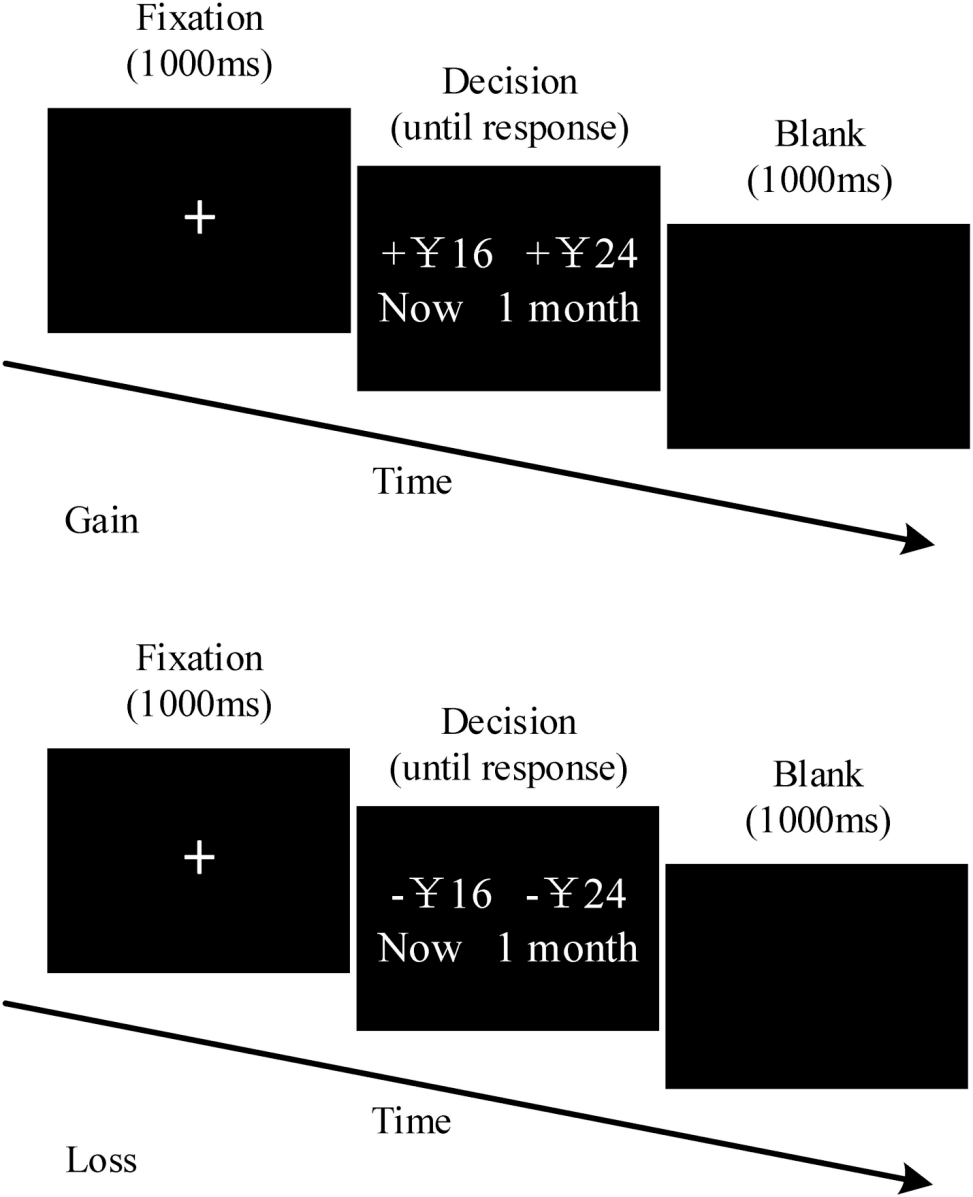
Trial structure for temporal discounting task of gains and losses. The gain session and the loss session had identical forms. There a 5-min interval between the gain session and loss session.

The experimental materials was described in the studies of McClure et al. (2004a) and Xu et al. (2009), which include a block of 84 gain trials (G-TD) and one with 84 loss trials (L-TD), as shown in Figure 1. Half of the participants performed the G-TD block first, while the other half conducted the L-TD block first. The order of both the trials within a block and the location (left or right) of the options (SS/LL) were randomly assigned. The SS alternative showed whether participants either obtained or lost the amount of money in that instance (1 month later), whereas the LL option indicated that the amount of money would be either obtained or lost after 1 month (or 2 months). The monetary value (CNY) of the SS option was taken from a Gaussian sequence with a mean of 50 and a standard deviation of 25, ranging from 13 to 120. The percent differences between the SS and LL options were selected from the following set: 5, 10, 15, 20, 35%, and 50%. G-TD and L-TD held the same delay and values, the only exception was the fact that a “+” indicated the gain and a “–” the loss.

The experiment was conducted in a small, electrically shielded room. The E-Prime software (Version 2.0) was used to display the stimuli. Briefly, after taking the electrode caps, participants were asked to concentrate on the computer display. The central sign “+”: was first presented for 1,000 ms, followed by the stimulus pictures. Successively, participants were instructed to press a button to indicate their choices (button “1” for left-side and “2” for right-side options). Thereafter, a blank screen appeared between two consecutive sequences for 1,000 ms. Participants were told prior to the experiment that one trial of their choices would be randomly selected as their “true appearance fees,” to ensure the participants' engagement in the decision-making task. Specifically, the fee would be given directly according to their selections in gain trials, whereas the fee would be CNY 150 (the maximum amount one could get) minus the result of the selection during loss trials. However, all the participants actual received the same amount (CNY 50) at the end of the study, regardless of the trial they selected. Furthermore, participants were required to take a practice round (4 trials) before the formal tasks. After the completion of the practice, participants could freely choose whether to enter the testing task or continue practicing, according to their understanding of the experimental task.

### EEG Recording and Preprocessing

The 64-lead electrode cap, extended by the international 10–20 system, was used in this experiment to record the EEG signals with the Scan 4.5 (NeuroScan Inc.) software. Moreover, two electrodes were placed on participants' right eye socket (1 cm above and 1 cm below) to record vertical electrooculography (VEOG). Two additional electrodes were located on the left and right eyes sides, near the temple, to record the horizontal electrooculogram (HEOG). The average reference was used to record signals. Furthermore, the band-pass filter was selected to be 0.05–100 Hz, while the sampling mode adopted the AC sampling (1,000 Hz sampling rate). All electrode impedances were maintained below 5 kΩ during the recording.

After data collection, the Curry 7 software (Russo et al., 2015) was employed to analyze the off-line data. The mean of the bilateral mastoids was re-referenced, whereas those VEOG data with amplitudes between −200 and 0 μV were automatically excluded. Successively, other electrodes with amplitudes surpassing ±100 μV were removed. The resulting signals were low-pass filtered at 30 Hz. The EEG data was cut by taking the 200 ms prior to and the 500 ms following stimulus onset as an epoch. In contrast, the 200 ms prior to the stimulus were used as a baseline.

Finally, three ERP components, viz. P200, N2, and P300, were extracted on the characteristics of the total average signal of all subjects. The P200 component, which mainly occurs in the prefrontal cortex, is a positive amplitude with a typical latency of 200 ms and is usually generated during the early stages of the decision-making process (Carretié et al., 2001, 2014). Furthermore, three electrodes, i.e., Fz, F1, and F2, were employed to measure the peaks appearing between 150 and 250 ms, defined as the P200 component. In contrast, the N2 component mainly appears in the frontal lobe region (Folstein and Van Petten, 2008) and the amplitude of the three electrodes, i.e., Fz, F1, and F2, in the following time windows, 250–350 ms after stimulus, were used to measure it. Finally, the P300 component was determined by electrodes Pz, P1, and P2, with the same time windows (250–350 ms after stimulus). Subsequently, both the context (gain vs. loss) and the electrodes were analyzed through repeated measures analysis of variances (ANOVAs). Finally, ANOVAs were conducted to analyze both the selection (SS vs. LL) and the electrodes.

### EEG Connectivity Analysis

After pre-processing and ERP processing, relatively pure EEG segmentation data were obtained for the analysis of brain functional connectivity. Specifically, while the weighted phase lag index (wPLI) analysis was used to measure the functional connectivity between electrodes (nodes), the standard phase lag index analysis quantifies the phase synchronization in two different time series by detecting non-zero phase difference coupling. The wPLI expands the PLI by additionally accounting for the magnitude of the phase difference between the two time series (Vinck et al., 2011; Lau et al., 2012; Cavanagh and Frank, 2014; Hardmeier et al., 2014). The phase lag analysis steps for each EEG signal were as follows: (1) EEG signals were subdivided according to different frequency bands (delta: 0.1~4 Hz; theta: 4.1~8 Hz; alpha: 8.1~12 Hz; beta: 12.1~25 Hz); (2) the wPLI of the EEG signal in each frequency band, for each condition, for each subject, were calculated using HERMES (Niso et al., 2013) software; (3) the appropriate threshold to maintain a 30% connection density in the connected network under each frequency band was confirmed (Achard and Bullmore, 2007; Bullmore and Bassett, 2011). In the present, the threshold proportional function of the BCT (Brain Connectivity Toolbox, Rubinov and Sporns, 2010) toolkit was employed to filter connections. Moreover, to ensure optimal network connectivity under the both gain and loss conditions, the threshold was set to 0.3 (i.e., retain the strongest connection of 30%). The connection value of each subject in the connection matrix was set to 1 when their absolute value was larger than the threshold. Other connection values in the connection matrix were set to 0.

### Graph Theoretical Analysis

Based on the binary network of the phase lag numerical transformation, the network's small-worldness index was adopted as the analysis index to compare the differences in the gain and loss contexts. Recent studies identified the brain to usually be a network with “small-world” properties, which is commonly thought to represent an optimal balance between globe integration and local segregation (Achard and Bullmore, 2007; Rubinov and Sporns, 2010; Pandit et al., 2013). Small-world networks represented by regular networks and random networks have both a high clustering coefficient (similarly to the former) and a short characteristic path (similarly to the latter) (Watts and Strogatz, 1998; Jin et al., 2012). On the one hand, clustering coefficient is the reflection of both the local integration ability and the grouping of the network (Rubinov and Sporns, 2010). We considered the average clustering coefficient of all nodes in the network as the clustering coefficient of the network (Onnela et al., 2005).

$$\begin{array}{l} C=\frac{1}{n}\underset{i\in N}{\sum }C_{i}=\frac{1}{n}\underset{i\in N}{\sum }\left[\frac{2E_{i}}{K_{i}\left(K_{i}-1\right)}\right] \end{array}$$

Where *E*_*i*_ is the number of ed**g**es actually existing in the neighbor node connected by node *i*, and *K*_*i*_ is the number of neighbor nodes connected by node *i*. However, on the other hand, the characteristic path length describes the ability of information transmission within the network, which is a global network feature, and reflects the strength of the functional integration of brain areas (Rubinov and Sporns, 2010). We first measured the shortest path between all the pairs of nodes in the network, while, the characteristic path length of the network is equal to the average of all shortest path lengths (Latora and Marchiori, 2001).

$$\begin{array}{l} L=\frac{1}{\frac{1}{n\left(n-1\right)}\underset{i\neq j\in N}{\sum }\frac{1}{d_{ij}}} \end{array}$$

Where *d*_*ij*_ is the shortest path length between node *i* and node *j*. To quantify the “small-world” property, a random network is treated as a reference. If a network has large cluster coefficients and similar characteristic path length compared with a random network (γ = *C*_*real*_/*C*_*random*_ ≫ 1, λ = *L*_*real*_/*L*_*random*_ ≈ 1, where the subscript *random* is a random network with the same amount of nodes and subscript *real* is a real network), the network belongs to the category of “small-world” networks. We used the method proposed by Humphries and Gurney (2008) to combine two metrics into a scalar $\sigma =\frac{\gamma }{\lambda }$ to determine the “small-world” characteristic. Specifically, when γ > 1, the network has “small-world” properties (Humphries and Gurney, 2008).

## Results

### Behavioral Data

#### The Proportion of the Chosen SS Options

**Figure 2:**
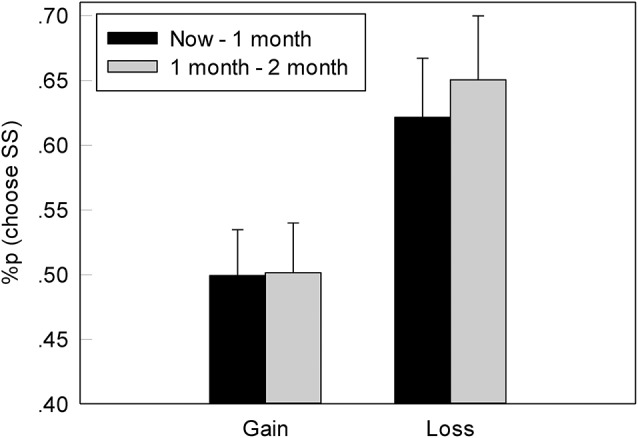
The mean percentage of choice for an immediate option of reward in G-TD and L-TD. Error bars denote standard error of the mean.

In both the G-TD and L-TD, participants were more inclined to choose the SS options, which is in line with our expectations. The proportions of the chosen the SS options at different times (now, 1 month, 1–2 months) based on both the gain and loss contexts were shown in Figure 2. Furthermore, repeated-measures ANOVA found a significant difference between the gain and loss contexts, *F*_(1, 22)_ = 4.759, *p* = 0.04, η_*p*_^2^ = 0.178. Specifically, the proportion of the chosen SS options in the L-TD was significantly higher than that in the G-TD. However, neither a significant difference at different times (now, 1 vs. 1–2 month), *F*_(1, 22)_ = 0.708, *p* = 0.409, η_*p*_^2^ = 0.031, nor an interaction between the context (gain vs. loss) and time was observed, *F*_(1, 44)_ = 0.664, *p* = 0.424, η_*p*_^2^ = 0.029.

#### The Discounting

**Figure 3:**
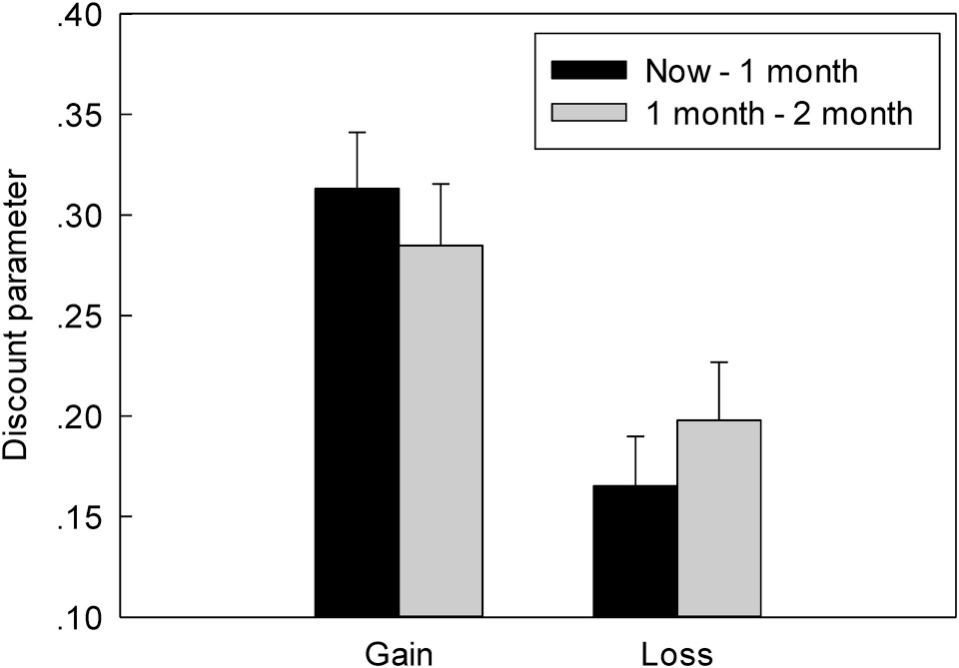
The discount rate of different conditions in G-TD and L-TD. Error bars denote standard error of the mean.

The more preference for the SS option, the higher discount rate in G-TD and the lower discount rate in L-TD was found. The indifference point was the turning point from the preferred SS option to the LL option, representing the point of no preference (i.e., the subjects' choice in the gain and loss contexts). We used the hyperbolic model (Mazur et al., 1987) to convert the indifference point to the subjects' discount, which was represented by *k*. The formula is as follows:

$$\begin{array}{l} V\;=\;A/\left(1+kD\right) \end{array}$$

where V is the value of the immediate option, A is the value of the delay option, and *D* is the delay time (for ease of calculation, here we used “month” as a unit of delay time). The discount parameter of the gain condition was greater than that of the loss condition (Figure 3). Furthermore, ANOVAs indicated a significant main effect of context, *F*_(1, 22)_ = 18.435, *p* < 0.001, η_*p*_^2^ = 0.456, as the discount parameter in the L-TD was significantly less than that in the G-TD. Finally, an absence of both the main effect on time, *F*_(1, 22)_ = 0.005, *p* = 0.946, η_*p*_^2^ = 0.001, and the interaction between context and time was observed, *F*_(1, 22)_ = 1.994, *p* = 0.172, η_*p*_^2^ = 0.083.

### ERP Results

#### P200

**Figure 4:**
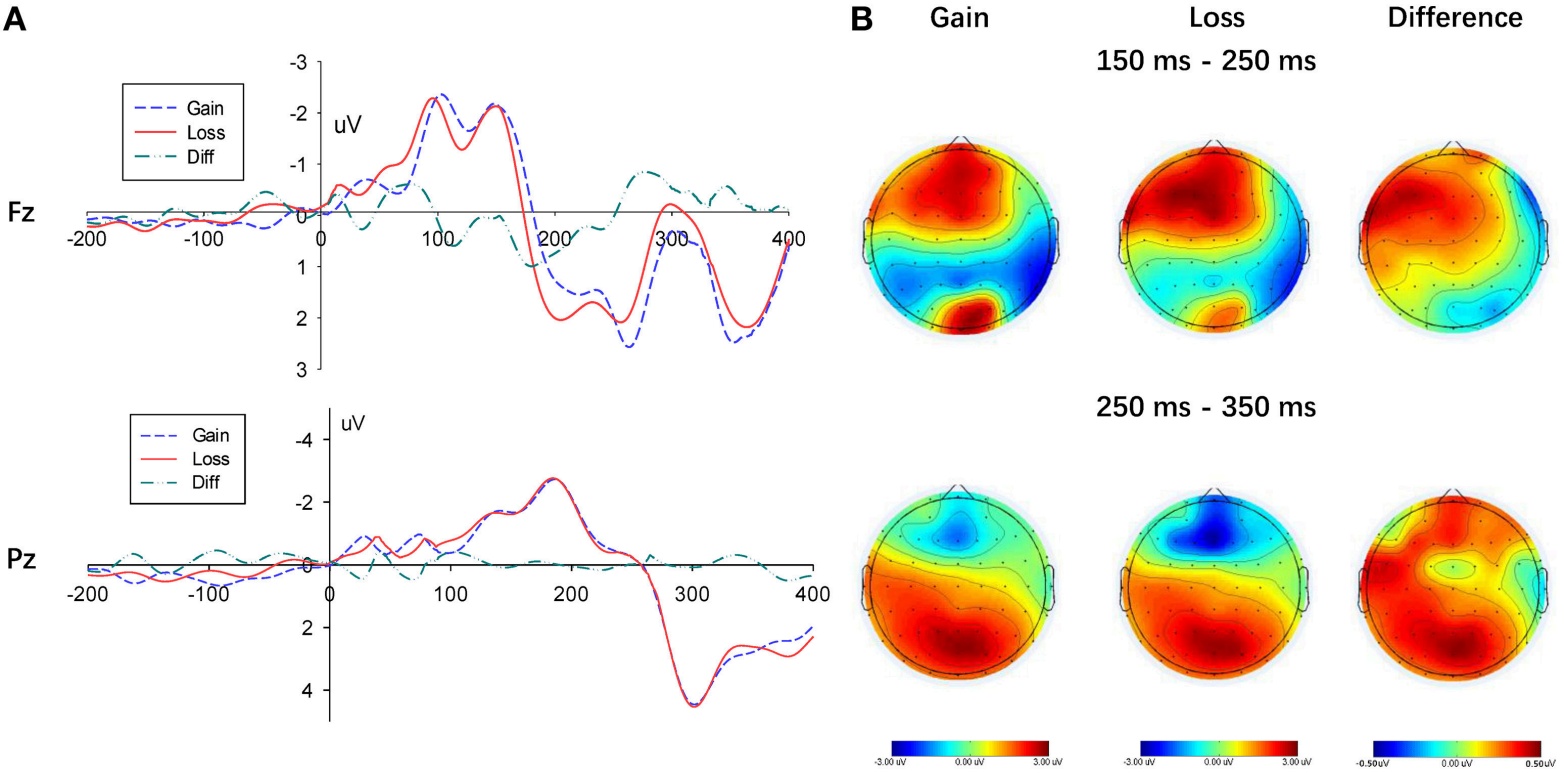
Results of the gain and loss conditions. **(A)** Representative example: ERP waveform in Fz and Pz; **(B)** topographic maps for P200 (150 ms-250 ms), N2 (250 ms-350 ms) and P300 (250 ms-350 ms).

The stimulus caused a discernable P200 component in the frontal area of the brain (measurement electrodes FZ, F1, and F2, measurement time window 150–250 ms). As shown in Figure 4A, its amplitude in the loss condition was greater than that in the gain context. Additionally, to compare the amplitude difference in the P200 component between the gain and loss conditions, the result valence was considered as an independent variable, while the average of the three electrode amplitudes was determined as the dependent variable. The repeated-measures ANOVA showed a significant main effect of context (gain vs. loss), *F*_(1, 22)_ = 5.443, *p* = 0.029, η_*p*_^2^ = 0.198, the amplitude of the P200 component in the gain condition was significantly smaller than in the loss condition. Neither a main effect on the electrode, *F*_(2, 44)_ = 2.560, *p* = 0.089, η_*p*_^2^ = 0.1, nor an interaction between the decision type and the electrode were found, *F*_(2, 44)_ = 0.061, *p* = 0.941, η_*p*_^2^ = 0.003.

**Figure 5:**
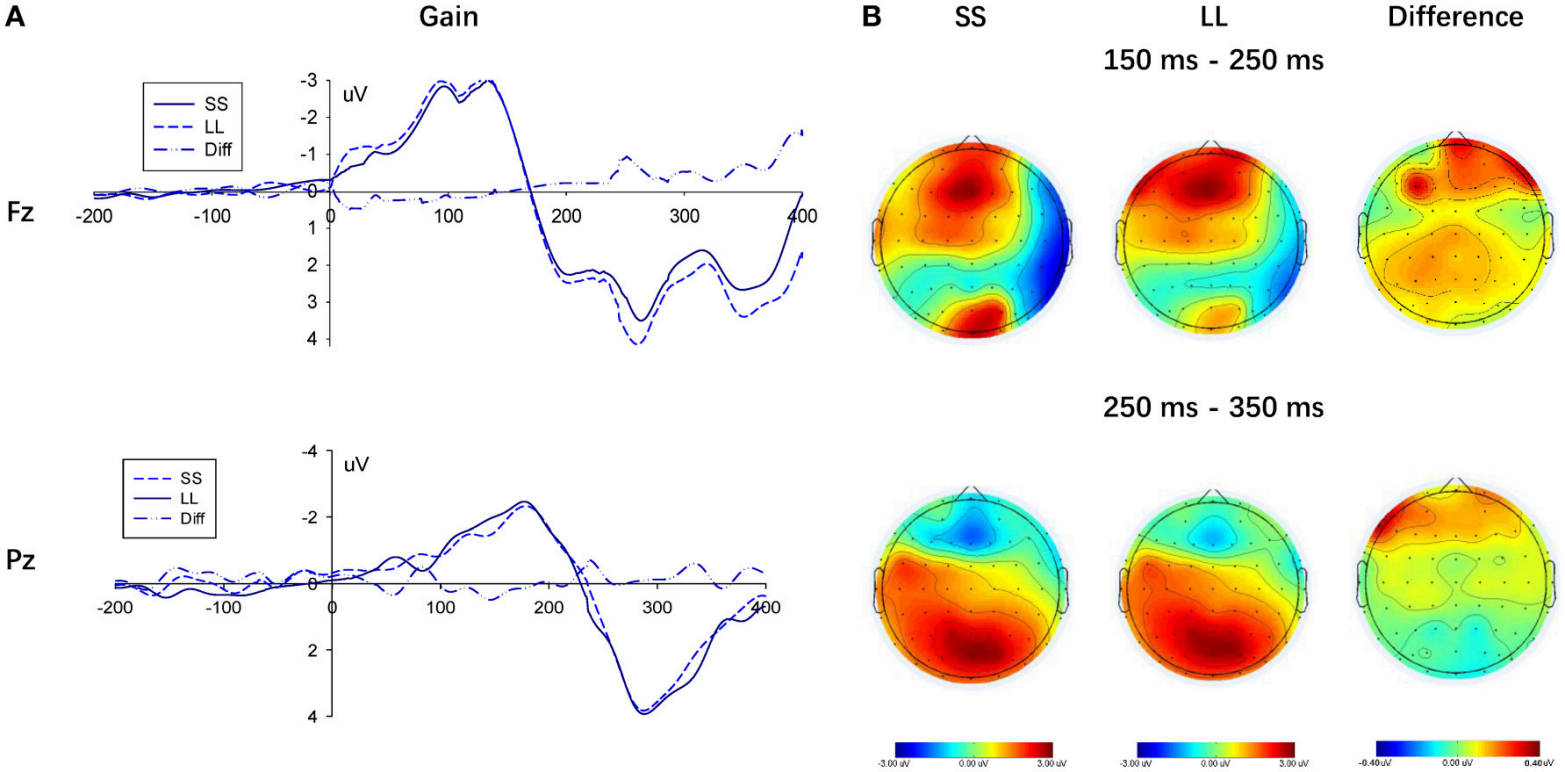
Results of selecting SS option and LL option in the gain condition. **(A)** Representative example: ERP waveform in Fz and Pz; **(B)** topographic maps for P200 (150 ms-250 ms), N2 (250 ms-350 ms) and P300 (250 ms-350 ms).

**Figure 6:**
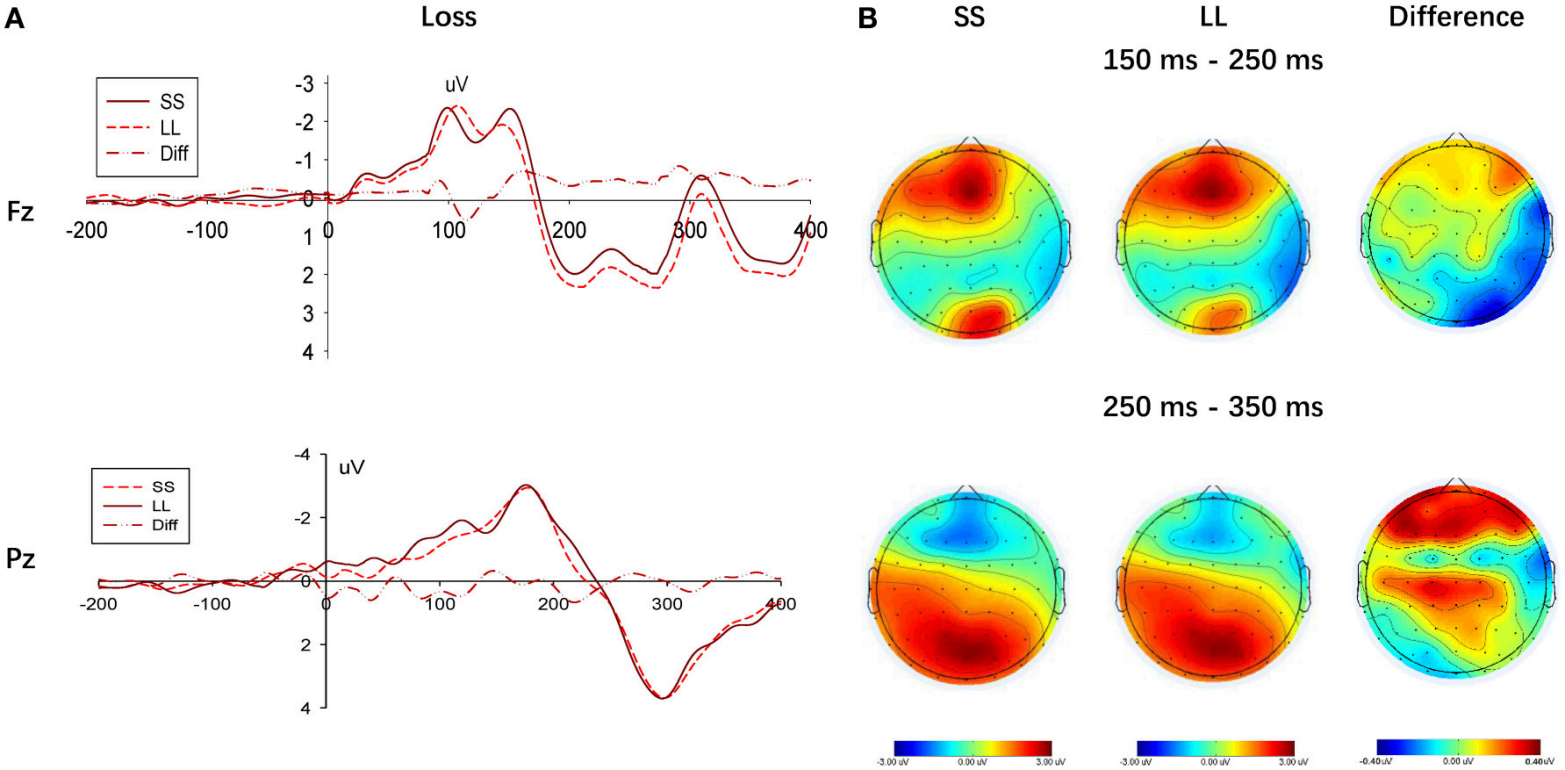
Results of selecting SS option and LL option in the loss condition. **(A)** Representative example: ERP waveform in Fz and Pz; **(B)** topographic maps for P200 (150 ms-250 ms), N2 (250 ms-350 ms) and P300 (250 ms-350 ms).

To compare the choice between the SS and LL options, we performed measurement analysis under the gain and loss conditions, respectively (Figures 5, 6). In the loss condition, the amplitude of the P200 component of the LL option was significantly greater than that of the SS option, *F*_(1, 22)_ = 6.087, *p* = 0.022, η_*p*_^2^ = 0.217, although a significant main effectin the gain condition was not observed, *F*_(1, 22)_ = 3.812, *p* = 0.064, η_*p*_^2^ = 0.148.

#### N2

Furthermore, three electrodes (i.e., Fz, F1, and F2) in the frontal region were selected to measure the N2 component (250–350 ms). A significant main effect of context (gain vs. loss) arose on the N2 amplitude, *F*_(1, 22)_ = 4.932, *p* = 0.037, η_*p*_^2^ = 0.183, given that amplitudes in the loss condition were significantly more negative than those in gain condition (see Figure 4). However, the N2 component did not show any significant difference at the electrodes, *F*_(2, 44)_ = 1.190, *p* = 0.287, η_*p*_^2^ = 0.051, while a significant interaction between context and electrodes was not found, *F*_(2, 44)_ = 0.631, *p* = 0.435, η_*p*_^2^ = 0.028.

In the frontal region, a negative wave was observed in the time window between 250 and 350 ms, as shown in Figure 5B. Although a main effect between choices (SS vs. LL) was not found in G-TD, *F*_(1, 22)_ = 0.297, *p* = 0.591, η_*p*_^2^ = 0.013, a significant difference in the N2 component was described when selecting either the SS or LL options in the L-TD, *F*_(1, 22)_ = 8.340, *p* = 0.009, η_*p*_^2^ = 0.275, given that the amplitudes for the SS options were significantly more negative than those for the LL options. Finally, the main effect of electrodes on the N2 amplitude, *F*_(2, 44)_ = 1.272, *p* = 0.272, η_*p*_^2^ = 0.055, and the interaction between electrodes and choices, *F*_(2, 44)_ = 0.664, *p* = 0.424, η_*p*_^2^ = 0.029, were not significant.

#### P300

In addition, three electrodes (i.e., Pz, P1, and P2) were selected in the occipital region to measure the P300 component (250–350 ms). A lack of significant main effect of context (gain vs. loss) on the P300 amplitude was found, *F*_(1, 22)_ = 2.714, *p* = 0.114, η_*p*_^2^ = 0.110, (see Figure 4). Moreover, the P300 component did not show any significant difference at the electrodes, *F*_(2, 44)_ = 2.856, *p* = 0.105, η_*p*_^2^ = 0.115, while a significant interaction between context and electrodes was also not found, *F*_(2, 44)_ = 1.659, *p* = 0.202, η_*p*_^2^ = 0.070.

Further analysis of the P300 components on the SS and LL options indicated that a main effect between the choices (SS vs. LL) was not present, regardless of the condition (i.e., gain or loss), *F*_(1, 22)_ = 0.279, *p* = 0.602, η_*p*_^2^ = 0.013, and *F*_(1, 22)_ = 0.015, *p* = 0.907, η_*p*_^2^ = 0.001, respectively (see Figure 6).

### Brain Network

#### Small World Attributes

**Figure 7:**
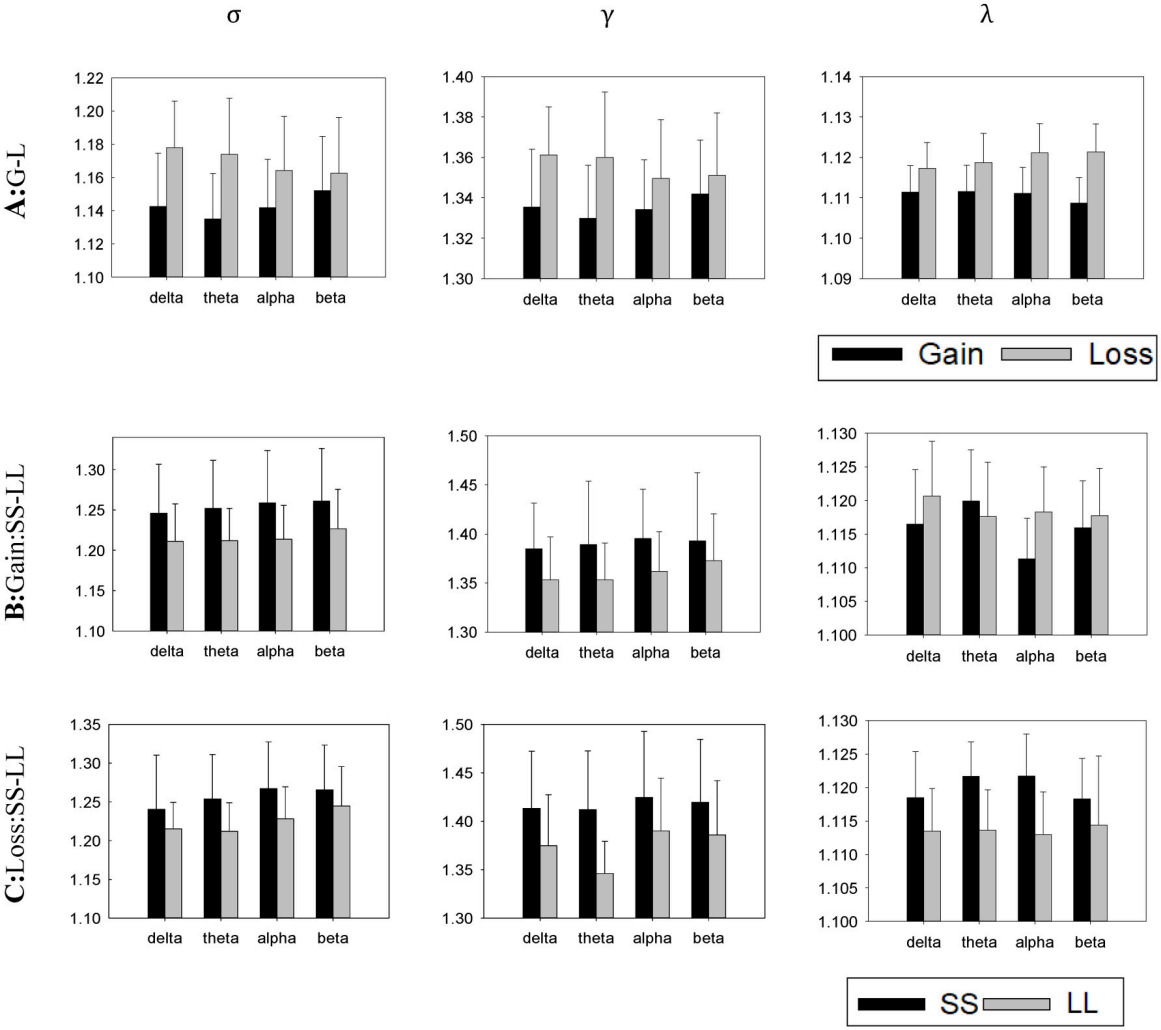
Averaged normalized small-worldness index (σ), clustering coefficient (γ) and characteristic path length (λ) of the brain networks for all task conditions (A, gain vs. loss; B, SS vs. LL in a gain condition; C, SS vs. LL in a loss condition). Error bars denote standard error of the mean.

Based on previous data processing, the small-worldness index (σ), clustering coefficient (γ), and characteristic path length (λ) were calculated in each frequency band using the above-mentioned formula. Figure 7 shows the histogram at each frequency band separately.

1. Delta band (δ: 0.1–4 Hz): A significant difference in σ, *t*_(22)_ = −3.303, *p* = 0.003, γ, *t*_(22)_ = −2.766, *p* = 0.011, and λ, *t*_(22)_ = −2.484, *p* = 0.021, was found for context. Furthermore, a follow-up analysis suggested that σ, γ and λ do not present any significant difference between choices (SS vs. LL) in the contexts of gain and loss (see Table 1 for details).
2. Theta band (θ: 4.1–8 Hz): A significant difference in σ, *t*_(22)_ = −4.025, *p* = 0.001, γ, *t*_(22)_ = −3.494, *p* = 0.002, and λ, *t*_(22)_ = −3.369, *p* = 0.001 was found for context. Furthermore, a follow-up analysis suggested that the LL choice showed smaller σ in both contexts, *t*_(22)_ = 3.429, *p* = 0.002, and, *t*_(22)_ = 2.302, *p* = 0.031, respectively. In contrast, an absence of significant difference in λ between choices in the different contexts was reported, whereas a significant difference in γ between the choices in the of loss context was observed, *t*_(22)_ = 5.197, *p* < 0.001, although the same was not valid for the gain context (see Table 1 for details).
3. Alpha band (α: 8.1–12 Hz): A significant difference in σ, *t*_(22)_ = −2.405, *p* = 0.025, and γ, *t*_(22)_ = −4.817, *p* < 0.001, was found for context. However, a significant difference of context was observed not for λ, *t*_(22)_ = 2.047, *p* = 0.053. Furthermore, a follow-up analysis suggested that the LL choice showed smaller σ for both the gain and loss contexts, *t*_(22)_ = 2.754, *p* = 0.012, and *t*_(22)_ = 2.771, *p* = 0.011, respectively (see Table 1 for details).
4. Beta band (β: 12.1–25 Hz): A significant difference in σ, *t*_(22)_ = −1.311, *p* = 0.204, and γ, *t*_(22)_ = −1.169, *p* = 0.225 was not found for context. However, a significant difference of contexts was observed for λ, *t*_(22)_ = −6.808, *p* < 0.001. Furthermore, a follow-up analysis suggested the σ, γ, and λ not to be significantly difference for the choices in both contexts (see Table 1 for details).

**Table 1:** Results of paired sample *T*-test (Context: Gain vs. Loss; Choice: SS vs. LL) on small-worldness index (σ), clustering coefficient (γ), and characteristic path length (λ).

**Frequency band**	**Condition**	**Small-worldness index (σ)**	**Clustering coefficient (γ)**	**Characteristic path length (λ)**
Delta band (δ)	Gain-Loss	*t*_(22)_ = −3.303 *p* = 0.003	*t*_(22)_ = −2.766 *p* = 0.011	*t*_(22)_ = −2.484 *p* = 0.021
	Gain: SS-LL	*t*_(22)_ = 2.014 *p* = 0.056	*t*_(22)_ = −1.897 *p* = 0.071	*t*_(22)_ = −1.844 *p* = 0.079
	Loss: SS-LL	*t*_(22)_ = 1.564 *p* = 0.132	*t*_(22)_ = 2.373 *p* = 0.027	*t*_(22)_ = 1.770 *p* = 0.091
Theta band (θ)	Gain-Loss	*t*_(22)_ = −4.025 *p* < 0.001	*t*_(22)_ = −3.303 *p* = 0.003	*t*_(22)_ = −3.679 *p* < 0.001
	Gain: SS-LL	*t*_(22)_ = 2.302 *p* = 0.031	*t*_(22)_ = −2.045 *p* = 0.053	*t*_(22)_ = 0.614 *p* = 0.546
	Loss: SS-LL	*t*_(22)_ = 3.429 *p* = 0.002	*t*_(22)_ = 5.197 *p* < 0.001	*t*_(22)_ = 1.974 *p* = 0.061
Alpha band (α)	Gain-Loss	*t*_(22)_ = −2.405 *p* = 0.025	*t*_(22)_ = −1.958 *p* = 0.065	*t*_(22)_ = −4.817 *p* < 0.001
	Gain: SS-LL	*t*_(22)_ = 2.754 *p* = 0.012	*t*_(22)_ = −1.890 *p* = 0.072	*t*_(22)_ = −3.807 *p* < 0.001
	Loss: SS-LL	*t*_(22)_ = 2.771 *p* = 0.011	*t*_(22)_ = 2.089 *p* = 0.048	*t*_(22)_ = 3.137 *p* = 0.005
Beta band (β)	Gain-Loss	*t*_(22)_ = −1.311 *p* = 0.204	*t*_(22)_ = −1.129 *p* = 0.255	*t*_(22)_ = −6.808 *p* < 0.001
	Gain: SS-LL	*t*_(22)_ = 1.978 *p* = 0.061	*t*_(22)_ = −1.114 *p* = 0.277	*t*_(22)_ = −0.151 *p* = 0.882
	Loss: SS-LL	*t*_(22)_ = 1.400 *p* = 0.175	*t*_(22)_ = 2.047 *p* = 0.053	*t*_(22)_ = 1.399 *p* = 0.176

To summarize, we found differences in the small-world network for the gain and loss contexts in the delta, theta, and alpha bands. Specifically, larger small-worldness index and clustering coefficient were observed in the loss condition, indicating that the brain network had stronger group characteristic. However, while in theta and beta bands, a significant difference in the characteristic path lengths of the context (gain vs. loss) was not seen, the same is not valid for the alpha band, suggesting that the brain network reported little difference in information transmission in different contexts. Finally, we described a smaller small-wordness index (σ) for the LL selections in the theta and alpha bands in the both contexts.

#### Functional Connection Network

**Figure 8:**
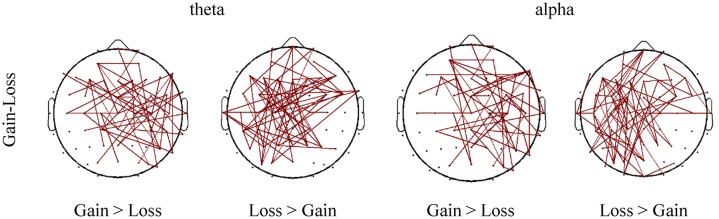
Differences of connectivity strength between different task conditions (gain vs. loss). Only the connections with absolute differences greater than 0.05 are shown in the figure.

To further characterize the differences between contexts and time delay on the brain network, the phase lag index matrix from the theta and alpha bands was selected and each pair of electrodes was compared by point-to-point *T*-test (0.05 was chosen as a reference value). Figure 8 shows the difference in connection strength in the two contexts for the theta and alpha bands.

Specifically, stronger cortical connections were observed in the theta band in the frontal-central area in the loss context, compared to those in the gain context. In contrast, the gain context showed stronger cortical connections in the right central region compared to the loss context.

With regards to the alpha band, the frontal lobe area presented stronger cortical connections in the loss context, whereas the gain context evoked stronger cortical connection in the right central and frontal-central areas.

## General Discussion

In the current study, both the brain electrophysiological activities and the discrepancy of brain network properties underlying intertemporal decision-making were investigated under the gain and loss framework using ERPs and graph theory analysis. Our behavioral results indicate a smaller discount rate in a loss context compared to a gain context, meaning that subjects preferred SS options. At the brain level, our findings reported both a greater P200 amplitude and a more negative N2 amplitude in the loss context, compared to the gain context. However, the P300 component was not different between the two contexts. Furthermore, the brain network showed a stronger small-worldness index in a loss context, which suggested that stronger emotions occurred when a loss decision was made. Finally, the network connectivity under the conditions of gain and loss in the alpha band were compared. Gain context-related (gain > loss) cortical connections were mainly focused on the right central and frontal-central area, whereas the frontal lobe region presented loss context-related (loss > gain) connections. This finding indicated that both the differences in the brain network between gain and loss decisions and the cortical connections in the frontal lobe region may be related to the representation of negative emotions. Taken together, our findings propose that loss decisions lead to stronger negative emotions, resulting in a stronger N2 component, a small-worldness index and evoking stronger cortical connections in the left parietal-occipital area.

The behavioral results reported that participants chose the SS options more frequently and present a smaller discount rate in the loss condition. These results are consistent with previous studies (Green and Myerson, 2004; Scheres et al., 2013). Considering that since subjects usually had loss aversion in loss contexts, they would attentively weight diverse elements before making decisions. This could be considered as a type of non-impulsive condition, while the time discounting rate decreases in the loss context. Moreover, some studies concluded that variations in time perception led subjects to take impulsive decisions (Zauberman et al., 2009). Compared to the gain condition, the loss context brought the subjects to change their perception of the delay time, thinking time cost was too high, and then to prefer SS options.

Furthermore, three vital waveforms P200, N2, and P300 components were discovered. The P200 component mainly appeared in the prefrontal area and posed an ERP component in the early stage of decision (Carretié et al., 2001). Previous studies proved the P200 component to be related to the complexity of the issue (Carretié et al., 2001, 2014). The way in which a stimulus would impact the distribution of decision makers' attention may be adopted to explain the reason behind the higher P200 amplitude see in a loss context, as opposed to a gain condition. In fact, the amplitude of the P200 component was associated with the way people pay attention, i.e., the more the attention, the larger amplitude. Under the loss condition, a larger amplitude was induced given that our test subjects distributed more attention to the loss context. As all the stimulus materials contained some kind of loss, subjects had to take more factors into consideration. Previous ERP studies demonstrated that the frontal P200 component appeared in the early stage of cognitive decision-making and that it was related to the initial assessment of stimulus and the rapid emotional feedback (Huang and Luo, 2006; Potts et al., 2006; Nikolaev et al., 2008; Boudreau et al., 2009; Yu et al., 2018). While making intertemporal decisions, the amplitude of the P200 component of the SS options was significantly smaller than that of the LL options, indicating an unconscious increased attention when choosing the LL options. It is possible that wider numerical differences in the LL options might results in greater attention (Gui et al., 2016).

In contrast, the N2 component usually appears in conflict decision-making, as larger amplitudes are found in conflict situations (Nieuwenhuis et al., 2003). Previous studies showed the performance of the N2 component to be positively related to the perception of conflicts (Folstein and Van Petten, 2008). In fact, higher risks posed harder decisions and greater conflicts evoked larger amplitudes of N2 component (Yang et al., 2007). Moreover, it was previously described that some brain areas related to negative emotions, including the insula, thalamus, and dorsal striatum, were strikingly activated in loss decisions (e.g., Xu et al., 2009). Recently, several studies reported a positive correlation between the amplitude of the N2 component and negative emotions, as higher N2 amplitudes were identified under negative conditions (e.g., Gehring and Willoughby, 2002; Nieuwenhuis et al., 2003; Yuan et al., 2007; Wu et al., 2014; Ma et al., 2015). In concordance, a larger N2 amplitude was observed in the loss decisions of the current study, probably given the greater risk and the more negative emotions found in loss decision-making. Considering that risk aversion in loss decision-making created more conflicts, the negative emotion of loss aversion impacted on the sensitivity of time distance. Additionally, a greater N2 amplitude was seen when selecting the SS option than LL option, which was consistent to previous studies (Peters, 2011; Gui et al., 2016). This phenomenon is likely related to the greater loss aversion stimulated by loss decisions. In fact, during the loss decisions, subjects were asked to pay their loss immediately after choosing SS options, which easily evoked negative emotions. Therefore, SS options required subjects to overcome an increased psychological conflict. While subjects tolerate smaller risks for the LL options, a greater risk for SS options (pay for loss immediately) was implied. In fact, the amplitude of N2 component was related to conflict control and may be one of the key EEG components for predicting intertemporal decisions.

With regards to the P300 components, they are typically considered to be associated with attention, evaluation, decision-making, and memory processes; whereas a reduction in their amplitude is seen for more difficult information transmission tasks (Polich, 2007; Chen et al., 2009). However, in contrast to previous studies (e.g., Gui et al., 2016), we failed to find larger amplitudes of the P300 component when choosing the SS option rather than the LL option. It is possible that the individual differences and the relative small sample size resulted lack of significant of the P300. It is worth noticing that we reported a larger small-worldness index for SS choosing, indicating a greater information processing in the brain. Further studies with a larger sample size may be needed to better address this issue.

The functional brain network displayed great discrepancies between the gain and loss contexts. The small world network was widely applied to investigations on the neural functional connectivity, as combining the respective topological advantages of both the regular network and random networks, it reflects the comprehensive evaluation of the concentration and transmission ability of a network (Rubinov and Sporns, 2010; Jin et al., 2012). Our results confirmed that the small-worldness network existed at the delta, theta, alpha and beta frequency bands, supporting the hypothesis that the functional brain network is a small-world network. Furthermore, the comparison between the four bands indicated that the loss context resulted in both a stronger small-worldness index and a larger clustering coefficient in the theta band (Sporns and Zwi, 2004). Our study found a stronger small-worldness index and a larger clustering coefficient in theta band too. This suggests that people need more resources to make loss compared to gain decisions. Interestingly, the same result was found in the alpha band, which played a great part in both the decision and cognition assessments (Kolev et al., 2001). The difficulty of loss decisions seemed to lead to stronger features linked with the small-worldness network. In addition, both a larger clustering coefficient and a longer characteristic path length were found, which is in line with the ERP results for the gain and loss. Increased communication and integration activities, along with more complex information processing, were described while making loss decisions.

After further analysis, a significant difference of the brain connecting networks between the gain and loss contexts was present, i.e., a stronger central connection appeared in the gain context, while strong connections appeared in right central and frontal-central areas in the loss conditions. These findings suggested that human brains are more sensitive to a potential loss, guided by negative emotions, such as fear and aversion, which influence the structure of the brain connections and alter the functional connections under the gain and loss conditions.

During gain decisions, both the SS and LL options exhibited a small-worldness index (σ) at all the four bands. Specifically, although this was observed mainly at alpha band, the σ of the SS selecting network was significantly larger than that of the LL selecting network. An increase in the small-worldness index indicated a greater information processing in the brain when selecting SS options. A comparison study on both immediate and delay rewards conducted by McClure et al. proposed that mOFC participates in both coding the relative values of various rewarding stimulus (O'Doherty, 2004) and responding to the stimulation value of monetary discounting (Winstanley et al., 2004).

In concordance, both the SS and LL options demonstrated small-world features at all the four bands during loss decisions. However, compared to gain decisions, loss decisions induced a greater small-worldness index, which might relate to the complexity of loss decisions. In fact, loss decisions evoked emotional reactions, including terror or anxiety (Breiter et al., 2001; Colin, 2005). Previous findings indicated that either expected or experienced loss would imply the activation of the brain areas associated with negative emotions, such as the insula (Breiter et al., 2001). Our results were consistent with such findings and suggested that subjects experience much greater negative emotions when facing immediate loss.

To conclude, the context-related differences in the neural networks underlying intertemporal decision-making were explored in the current study by using both ERPs and network graph analysis. Our findings indicate the main difference between loss and gain decisions to be the influence of negative emotions. In fact, the N2 component, which is triggered by negative emotions, was reported as the key contrast seen between gain and loss decisions in the neural components. Finally, our results revealed small-worldness to be another crucial index reflecting the difference between such decisions. Taken together, our findings extended the available knowledge on the network analysis behind intertemporal decision-makings.

## Author Contributions

LZ and FH conceived and designed experiment. LZ, QZ, and ZS performed the experiment. ZS and HC analyzed data. LZ, LX, FH, ZS, and QZ wrote manuscript. All authors edited and/or approved the manuscript.

### Conflict of Interest Statement

The authors declare that the research was conducted in the absence of any commercial or financial relationships that could be construed as a potential conflict of interest.
